# Coloration in Flow: The Potential of *In Situ* Coloration of Casein Fibers to Mitigate Environmental Impact of Traditional Dyeing Methods

**DOI:** 10.1021/acssuschemeng.3c07437

**Published:** 2024-01-29

**Authors:** Joseph A. Houghton, Alenka Tidder, Marie Stenton, Richard S. Blackburn

**Affiliations:** † Leeds Institute of Textiles and Colour, School of Design, University of Leeds, Leeds LS2 9JT, United Kingdom; ‡ Keracol Limited, Nexus, Discovery Way, Leeds LS2 3AA, United Kingdom; ¥ London College of Fashion, University of the Arts London, London E20 2AR, United Kingdom

**Keywords:** coloration, dyeing, natural dyes, fibers, food waste, anthocyanins, valorization, sustainable processes, wet-spinning, regenerated protein fibers

## Abstract

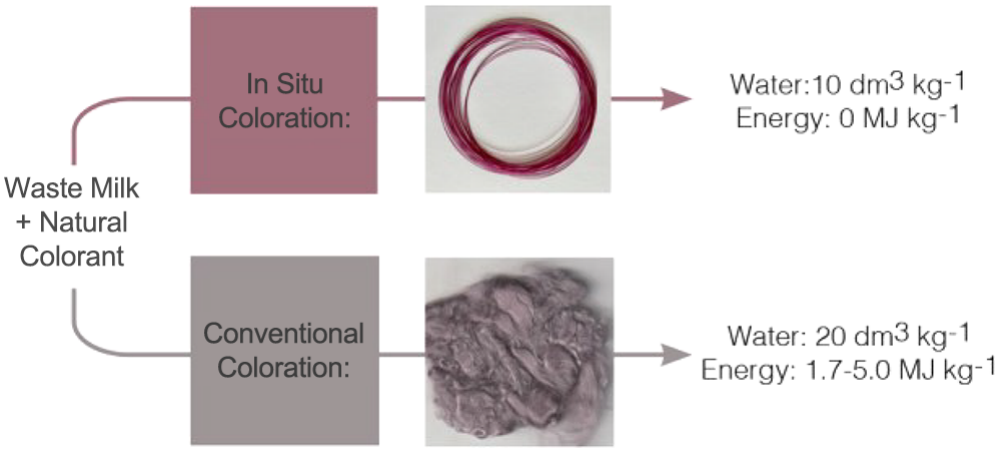

The environmental impact of the textiles and food industries can no longer be ignored, and while combining natural protein-based fibers with natural colorants, each derived from food waste, has the potential to offer increased sustainability based on a circular economy, it fails to address other environmentally detrimental textile production steps, such as coloration. This work explores the potential of a new, novel method for *in situ* coloration of regenerated protein fibers using an anthocyanin-based natural dye, used within the wet-spinning process, to reduce the environmental impact of the dyeing process. It is observed that similar or improved dye sorption and much improved 3D sustainability metrics (energy and material intensity) can be achieved through dyeing of casein fibers in flow, with higher color strength (*K*/*S*_*λ*__max_ = 2.5) observed under milder conditions (room temperature, 10 s) compared to conventional dyeing (*K*/*S*_*λ*__max_ = 1.0 at 40 °C, 30 min; *K*/*S*_*λ*__max_ = 2.7 at 80 °C, 30 min). Energy intensity calculations show conventional dyeing requires 1.7–5.0 MJ kg^–1^ fiber, depending on the dyeing temperature for experiments performed in this paper and up to 13.4 MJ kg^–1^ fiber for examples in the literature. Using coloration in flow, energy intensity is negligible showcasing a vast improvement in energy-based metrics. The *in situ* experimental method showed a material intensity of 10.2 compared to 21.2 of the conventional method explored and up to 40.2 for examples in the literature, making the process in flow far less material intensive than conventional coloration methods, with additional potential for further material savings due to the recycling potential of the dyebath, which does not require auxiliary dyeing chemicals. Space time yield calculations showed that the productivity of the proposed method in flow is much higher (182.4 g L^–1^ h^–1^) compared to the conventional batch process (33.3–60.0 g L^–1^ h^–1^).

## Abstract 2

Exploration of novel in flow coloration method for regenerated protein fibers using food waste-derived natural dyes toward a lower environment impact for coloration of fibers.

## Introduction

Textile production is one of the most environmentally damaging industries on the planet. Global annual fiber production was over 113 million tonnes (Mt) in 2021 and is predicted to increase to 149 Mt by 2030.^1^ This increase has a direct impact on the amount of raw materials, energy, and water required for the textile industry with the potential for catastrophic environmental ramifications.

By the implementation of circular design principles, there is potential to help alleviate the issues presented by the vast fiber production of the modern world and reduce the impact of waste from another environmentally important sector: the food industry. Approximately one-third of all food produced in the world is wasted, representing a vast, underutilized feedstock for sustainable chemicals and materials.^2^ Regenerated fibers from waste is an area of active research with multiple biopolymers being explored, including the utilization of waste protein to create regenerated protein fibers (RPFs). This approach was used during the world wars to help alleviate material shortages, but there is modern day potential with RPFs offering solutions to global food waste and the environmental impact of fiber production.^3,4^

The textile coloration industry is being scrutinized for its efforts to become more environmentally benign, with regulatory bodies and consumers demanding evidence of reduced energy and water usage within dye phases, as well as increased interest in nonsynthetic dyes.

Anthocyanins are a group of polyphenolic compounds occurring within nature and responsible for pink, red, purple, violet, and blue coloration in fruits, vegetables, and flowers. They are nontoxic, water soluble, and plentiful within many food waste streams, such as pomace from juice and wine production. These compounds are of increasing interest to the sustainable color chemist as a potential substitute for synthetically derived dye compounds.^5−8^

Spin or dope dyeing has been identified as a more sustainable option to produce colored fibers through either melt or wet-spinning.^9^ This process has its limitations as only one color of fiber can be produced from the polymer “dope”, so the technology is only applied to colors with a large market demand. The economic and process development hurdle of generating new and different colors is large.

During wet-spinning, the dissolved polymer “dope” is extruded into a coagulation bath containing an antisolvent, causing the polymer to solidify into the fiber. This is not instantaneous, and the fiber does not finish forming until it is fully dry. This intermediate form, referred to as “never-dried” fiber, is characterized by a more “open” polymer network caused by the remaining presence of polymer dissolution solvent within the polymer matrix. There is potential to dye the fibers during this phase through inclusion of the dye into the treatment baths of the still-forming fiber, where the dye molecules diffuse more easily through the polymer network. Incorporating the dyeing step into fiber production could reduce the energy and materials required to dye the fibers and be more flexible than dope dyeing.

An *in situ* dyeing process utilizing a novel, modular, lab-scale wet-spinning rig was created to produce kilometer quantities of food waste-derived casein monofilament, dyed with anthocyanins in flow. This was compared to conventionally dyed casein fibers.

## Experimental Section

### Materials

Pure casein from bovine milk and all chemicals were obtained from Sigma-Aldrich. Commercial casein fiber sliver was purchased from George Weil, U.K. Blackcurrant (*Ribes nigrum* L.) pomace was obtained from A&R House, U.K. The dried blackcurrant extract dye powder was produced using the method presented by Farooque et al.^7^

### Conventional Dyeing Process

Casein fibers were dyed at pH 2, from 40 to 80 °C, in an aqueous solution with 5% omf (on mass of fiber) blackcurrant dye powder using a liquor-to-fiber ratio of 20:1 using the methodology presented by Blackburn et al.^8^ Full details can be found in the Supporting Information (SI).

### In-Flow Dyeing Process

**Figure 1:**
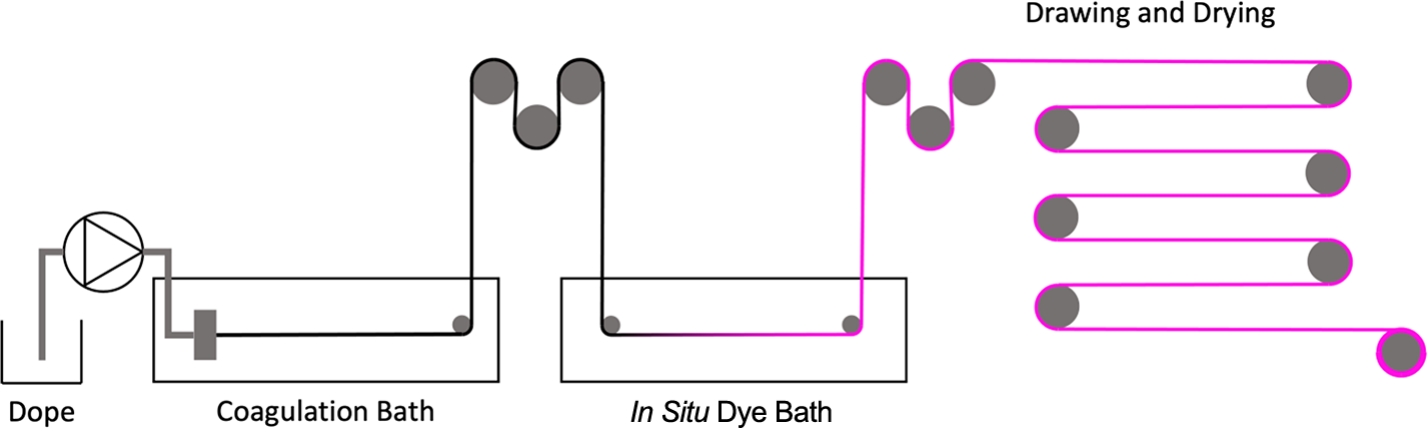
Simplified diagram demonstrating the combined wet-spinning and *in situ* coloration processes for casein fibers.

The *in situ* dyeing process involved using a novel, lab-scale, modular wet-spinning rig fabricated in-house to wet-spin casein fibers and then immediately dye the still forming fiber in a secondary coloration bath in a flow method. Figure 1 shows a simplified schematic diagram of this process. Full methodology, along with a video (Video S1) showing the process, can be found in the SI.

### Color Measurement

Conventionally dyed fibers and fibers dyed in flow were measured using a Datacolor 500 color spectrophotometer as described by Blackburn et al.^8^ to determine the color strength at maximum wavelength of absorption (*K*/*S*_*λ*__max_) and fiber color. Full details can be found in the SI.

### Sustainability Metrics

Key 3D sustainability metrics energy intensity and material intensity were selected based on the work of Martins et al.^10^ and as used by Xu et al.^11^ in their exploration of dyeing PLA. Because of the novel *in situ* dyeing methodology and its similarities to the continuous nature of flow chemistry, a commonly used metric used in flow chemistry, space time yield (STY), was also used as a measure of productivity in g L^–1^ h^–1^. These metrics were used to compare the novel coloration in flow method to the conventional dyeing method described herein and also to compare with literature examples of dyeing casein fibers with natural dyes^12^ and synthetic dyes.^13^ Full descriptions of the calculations and assumptions made can be found in the SI.

## Results and Discussion

### Colorimetric Analysis

**Figure 2:**
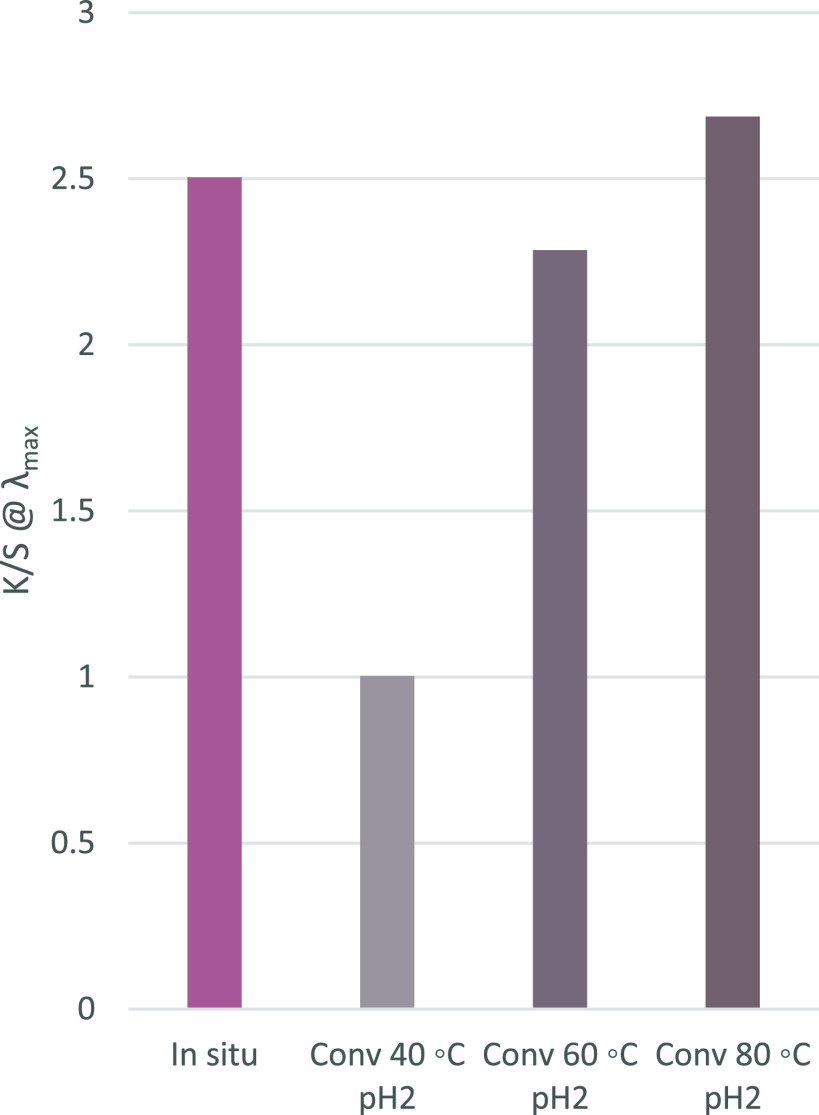
Comparison of color strength (*K*/*S*_*λ*__max_) between *in situ* and conventional methods. The color of the column represents the color of the dyed fiber.

Quantitative colorimetric work was carried out on the commercially sourced, conventionally dyed casein fibers and the *in situ* dyed fibers produced. From dyeing results (Figure 2), it is seen that a similar or greater color strength (*K*/*S*_*λ*__max_) is achieved from the process in flow under ambient conditions compared to conventional dyeing at elevated temperatures, which is unsurprising when the fiber morphology is considered. With conventionally dyed fibers, fiber morphology is compact, and elevated temperatures are required to swell the fiber to allow dye diffusion into the outermost layers. The process in flow means the fiber is dyed seconds after formation while its structure is still very open, allowing easy and swift diffusion of the solution throughout the fiber under milder conditions.

As reported by Blackburn et al.,^8^ the form of the anthocyanin heavily influences the resulting color of the dyed fiber. Anthocyanins have three forms, each with distinct colors, depending on the pH of dyeing solution: <pH 3, flavylium cation (**AH**^+^, red); pH 3–7, quinonoidal base (**A**, purple); pH > 7, anionic quinonoidal base (**A**^**–**^, blue). As the pH of the dyebath within the *in situ* dyeing process was highly acidic (due to the required conditions for wet-spinning of casein) the anthocyanin is likely 100% red **AH**^+^ form. The pH of the conventional dyeing was set at pH 2 to ensure 100% **AH**^+^ form and to allow for direct comparison. The fiber dyed in flow exhibits a much greater a* value in CIELab color analysis, indicating a shift toward red compared to the conventionally dyed samples; this is represented in Figure 2, with the color of the dyed fiber denoted by the color of the bar. It is theorized that this is due to much-improved dye penetration during the *in situ* process. After conventional dyeing, during the exhaust process, some of the anthocyanin molecules on the surface of the fiber are likely converted to the neutral or anionic forms of the molecule (characterized by a shift toward a blue color) meaning that the overall hue of the fiber also shifts toward blue. However, anthocyanin molecules diffused inside the fiber are theoretically protected from the aqueous environment and, therefore, retain their pink color. This is further evidenced by an observed shift toward red as the temperature of dyeing is increased, due to the greater dye diffusion found at elevated temperatures.

### Process Analysis via Sustainability Metrics

Because of the ease of dye diffusion into “never-dried” casein fibers during the wet-spinning process, it was predicted, and proven, that much milder conditions, reduced material usage, and vastly reduced dyeing times could yield similar or better results in terms of color intensity. This is beneficial from a process-intensification standpoint, as well as improving the sustainability metrics in the dyeing process. Some reasonable calculations for the material intensity, energy intensity, and productivity (as calculated through STY) for each experimental process (along with examples from literature) have been calculated and are summarized in Table 1; full calculations and assumptions made can be found in the SI.

**Table 1:**
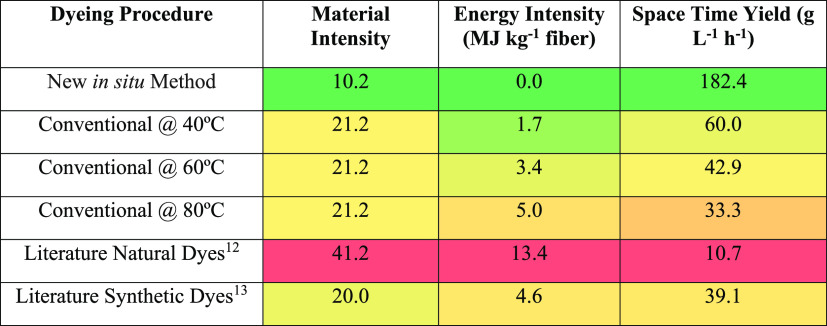
Overview of Sustainability Metrics for Conventional Dyeing vs Coloration in Flow from Both Experimental and Literature Values, with Color Scale Indicating Sustainability Preference

As the process in flow is unheated (room temperature), dyeing energy intensity can be assumed to be negligible, in keeping with literature conclusions for the reduction of energy usage in the LCA of dope-dyeing systems.^9^ Overall energy intensity of the conventional dyeing process used herein varied from 1.7 to 5.0 MJ kg^–1^ with varying temperature and was up to 41.2 MJ kg^–1^ for literature examples; in comparison, the energy intensity for coloration in flow is effectively zero. Material intensity for conventional dyeing processes (21.2 to 41.2) is substantially higher than for the *in situ* process (10.2). The productivity of the process reduces with increasing fiber:liquor ratio (as more volume is required to make the same mass of colored fiber) and with elevated temperature (as the time taken to reach the temperature increases). The conventional batch methods used herein show STYs of 33.3–60.0 g L^–1^ h^–1^, with STY as low as 10.7 g L^–1^ h^–1^ in literature methods, all of which are far lower than for the novel flow method (182.4 g L^–1^ h^–1^). All three metrics demonstrate that the coloration method in flow is an improvement over conventional exhaust dyeing processes in terms of energy intensity, material intensity, and productivity.

Additionally, the potential to recycle the dyebath is far greater with the *in situ* process compared to the conventional process. In the conventional process, the exhausted dyebath still exhibits intense color, presenting an inherent dye waste issue for effluent. Dyebath recycling in the conventional process is complicated by the auxiliaries chemicals present, which is not an issue for the *in situ* process. Within the conventional dyeing presented in this work, pH modifiers were the only auxiliaries used, but in industrial dyeing, there would be many more: dispersion agents, wetting agents, leveling agents, etc., which would lead to complications for dyebath recycling.^14^ Due to the increased dye penetration observed in the *in situ* process, these auxiliaries are likely not required, and the process, as it is continuous, exhibits much greater potential for bath recycling. If dye concentration was monitored throughout the spinning process (e.g., using UV–vis spectroscopy) and “topped up” when it reached unacceptably low levels, the dyebath could potentially be recycled indefinitely. In practice, this would likely not be possible as eventually the pH would be altered by any residual solvation agent present in the fiber, but as the dyeing step comes after the initial coagulation step (during which most of the polymer solvent is removed), this effect is likely to be small.

One major disadvantage of dope dyeing is the need to shut down and completely clean the entire spinning system if a new fiber color is required due to the colorant being added into the polymer dope and therefore present throughout the system. The novel *in situ* process completely removes the need for this as an uncolored polymer dope is used with an *in situ* dye bath, which could easily be swapped out for a different bath with a different coloration agent as required, making the *in situ* process more widely applicable to the coloration industry than dope dyeing.

### Limitations of the Coloration In-Flow System

Initial evidence indicates improvements that fiber coloration in flow could have over conventional dyeing systems, but there are limitations to consider when designing such a system. Dyeing conditions are limited to the fiber spinning process conditions. As the fiber has not yet finished solidifying, it is susceptible to any solvent and pH too similar to the original solvation agent: dyeing conditions must be suitable to keep the fiber solid during the process. This would limit the dyes that could be used within the process as they would have to be effective under, and resistant to, these conditions. This issue could potentially be circumvented by adding a cross-linking step before dyeing, but this would complicate the process. It also limits the conditions to those applicable to wet-spinning. Blackburn et al.^8^ reported that the optimal pH for dyeing of casein fibers with anthocyanins was pH 4, represented by a 60:40 ratio between the **A** and **AH**^**+**^ forms, respectively, but this was not possible in this particular in-flow process. So all comparisons within this paper are with dyeing at pH 2 (closest to the conditions used *in situ* and 100% **AH**^**+**^). For fibers dyed under optimal conditions using conventional exhaust processes (pH 4, 80 °C, 30 min), *K*/*S*_*λ*__max_ was 3.5, slightly higher than observed for the *in situ* process; however, for exhaust dyed fibers at lower temperatures (pH 4, 40 °C), closer to those of the *in situ* process (room temperature), lower *K*/*S*_*λ*__max_ (1.5) was observed, indicating that the *in situ* process is far more effective at lower temperatures (and shorter dyeing times) even when exhaust dyed at optimal pH. In other work, it was observed that color strength of proteinaceous fibers (hair) dyed with anthocyanins increases with increasing dye loadings, up to *K*/*S*_*λ*__max_ of 11.7,^15^ implying that the limit for color strength through dye penetration has not been reached in the work herein. Therefore, there is potential for the color strength of *in situ* dyed fibers to be increased above those achieved with the optimal exhaust dyeing conditions by increasing the dye loading in the process.

## Conclusions

This study has explored the potential for *in situ* dyeing of wet-spun fibers as a less environmentally impactful method of coloration. Including a coloration bath in the wet-spinning process alleviates many known issues around dope dyeing while dramatically improving key sustainability metrics such as energy intensity, material intensity, and productivity. The evidence from the colorimetric analysis of the produced fibers indicates *in situ* dyeing is more effective, likely due to the ease of dye penetration in the forming fiber’s more open polymer network. This allows for more effective dyeing under gentler conditions and could also potentially remove the need for dye auxiliaries, making the recycling of the dyebath by simple addition of more dye a possibility. There is much research to do in developing this proof-of-concept study to industrial relevance, but the possibilities for reducing the environmental footprint of the textile industry is huge.

## Supporting Information Available

Video S1: Novel *in situ* coloration method described herein (MOV)
Overview of experimental methodology and metric calculations and assumptions made (PDF)

## Supplementary Material

sc3c07437_si_001.mov

## References

[ref1] Preferred Fiber Materials Market Report, 2022. Textile Exchange. https://textileexchange.org/app/uploads/2022/10/Textile-Exchange_PFMR_2022.pdf (accessed 2023–10–20).

[ref2] de Los Mozos E. A.; Badurdeen F.; Dossou P.-E. Sustainable Consumption by Reducing Food Waste: A Review of the Current State and Directions for Future Research. Procedia Manuf 2020, 51, 1791–1798. 10.1016/j.promfg.2020.10.249.

[ref3] Stenton M.; Kapsali V.; Blackburn R. S.; Houghton J. A. From Clothing Rations to Fast Fashion: Utilising Regenerated Protein Fibres to Alleviate Pressures on Mass Production. Energies (Basel) 2021, 14 (18), 5654 10.3390/en14185654.

[ref4] Stenton M.; Houghton J. A.; Kapsali V.; Blackburn R. S. The Potential for Regenerated Protein Fibres within a Circular Economy: Lessons from the Past Can Inform Sustainable Innovation in the Textiles Industry. Sustainability 2021, 13 (4), 2328 10.3390/su13042328.

[ref5] Wathon M. H.; Beaumont N.; Benohoud M.; Blackburn R. S.; Rayner C. M. Extraction of Anthocyanins from Aronia Melanocarpa Skin Waste as a Sustainable Source of Natural Colorants. Coloration Technology 2019, 135 (1), 5–16. 10.1111/cote.12385.

[ref6] Tidder A.; Benohoud M.; Rayner C. M.; Blackburn R. S. Extraction of Anthocyanins from Blackcurrant (*Ribes nigrum* L.) Fruit Waste and Application as Renewable Textile Dyes. In Proceedings of the BIOColours Conference 2018; Leeds, 2018. 10.1021/acs.jafc.8b01044 29808681

[ref7] Farooque S.; Rose P. M.; Benohoud M.; Blackburn R. S.; Rayner C. M. Enhancing the Potential Exploitation of Food Waste: Extraction, Purification, and Characterization of Renewable Specialty Chemicals from Blackcurrants (Ribes Nigrum L.). J. Agric. Food Chem. 2018, 66 (46), 12265–12273. 10.1021/acs.jafc.8b04373. 30412401

[ref8] Blackburn R. S.; Houghton J. A.; Stenton M.; Tidder A. A Dye-Fibre System from Food Waste: Dyeing Casein Fibres with Anthocyanins. Coloration Technology 2023, na 10.1111/cote.12718.

[ref9] Terinte N.; Manda B. M. K.; Taylor J.; Schuster K. C.; Patel M. K. Environmental Assessment of Coloured Fabrics and Opportunities for Value Creation: Spin-Dyeing versus Conventional Dyeing of Modal Fabrics. J. Clean Prod 2014, 72, 127–138. 10.1016/j.jclepro.2014.02.002.

[ref10] Martins A. A.; Mata T. M.; Costa C. A. V; Sikdar S. K. Framework for Sustainability Metrics. Ind. Eng. Chem. Res. 2007, 46 (10), 2962–2973. 10.1021/ie060692l.

[ref11] Xu S.; Chen J.; Wang B.; Yang Y. Sustainable and Hydrolysis-Free Dyeing Process for Polylactic Acid Using Nonaqueous Medium. ACS Sustain Chem. Eng. 2015, 3 (6), 1039–1046. 10.1021/sc500767w.

[ref12] Benli H.; Bahtiyari M. İ. Dyeing of Casein Fibers with Onion Skin-Based Natural Dye Sources after Ozonation. Ozone Sci. Eng. 2018, 40 (2), 141–147. 10.1080/01919512.2017.1341300.

[ref13] Choi J.; Kim M. Dyeing Characteristics of Casein Protein Fiber with Acid Dyes and Reactive Dyes. Textile Coloration and Finishing 2008, 20 (5), 14–22. 10.5764/TCF.2008.20.5.014.

[ref14] Allègre C.; Moulin P.; Maisseu M.; Charbit F. Treatment and Reuse of Reactive Dyeing Effluents. J. Membr. Sci. 2006, 269 (1–2), 15–34. 10.1016/j.memsci.2005.06.014.

[ref15] Rose P. M.; Cantrill V.; Benohoud M.; Tidder A.; Rayner C. M.; Blackburn R. S. Application of Anthocyanins from Blackcurrant (Ribes Nigrum L.) Fruit Waste as Renewable Hair Dyes. J. Agric. Food Chem. 2018, 66 (26), 6790–6798. 10.1021/acs.jafc.8b01044. 29808681

