# Supplementary material for: Coloration in Flow: The Potential of In Situ Coloration of Casein Fibers to Mitigate Environmental Impact of Traditional Dyeing Methods
Source: ACS Sustain Chem Eng. 2024 Jan 29;12(6):2130–4. doi: 10.1021/acssuschemeng.3c07437 (PMC10865441; doi:10.1021/acssuschemeng.3c07437)
Supplement: Supplementary file 2 — sc3c07437_si_002.pdf [file sc3c07437_si_002.pdf]

Coloration in Flow: The Potential of *In Situ* Coloration of Casein Fibers to Mitigate Environmental Impact of Traditional Dyeing Methods

Joseph A. Houghton[†], Alenka Tidder[‡], Marie Stenton[¥], Richard S. Blackburn[†‡]*

† - Leeds Institute of Textiles and Colour, School of Design, University of Leeds, Leeds, LS2 9JT, UK

‡ - Keracol Limited, Nexus, Discovery Way, Leeds, LS2 3AA, UK

¥ - London College of Fashion, University of the Arts London, London, E20 2AR, UK

*Email: j.a.houghton@leeds.ac.uk

Number of Pages: 10

Number of Tables: 3

Number of Figures: 0

Supplementary Information

Conventional Dyeing Process

Casein fibers were dyed in an aqueous solution with 5% omf (on mass of fibre) blackcurrant dye powder using a liquor to fibre ratio of 20:1 by mass. Dyeing was performed at pH 2 adjusted using formic acid. Fibers were introduced to the dyebath at room temperature and then, using an Ugolini Redkrome II dyeing machine, the temperature was increased at a rate of 1 ˚C min^{-1} until the dyeing temperature was achieved, the samples were then held at this temperature for 30 mins. The dyeing temperatures explored were 40, 60 and 80 ˚C. Once the samples had been allowed to cool, they were rinsed in cold water and dried under ambient conditions prior to analysis.

In-Flow Dyeing Process

The *in-situ* dyeing process produced wet-spun casein fibers using a bespoke modular wet-spinning rig designed and fabricated in-house. The process involved the pumping of a spinning dope (22.5 wt% casein and 1.5 wt% NaOH in aqueous solution and aged for 16 h) through a 21G spinneret at a flow rate of 0.5 mL min^{-1} into a 500 mL aqueous coagulation bath containing 10 wt% H_2SO_4, 12 wt% $MgSO_4$ and Na_2SO_4. The extruded fiber was kept under the surface of the coagulation solution for the length of the bath (25 cm) followed by uptake at a velocity of 1.25 m min^{-1}. The fiber was then fed into a 500 mL aqueous dyebath containing 5 wt% H_2SO_4 and 1 wt% blackcurrant powder and kept under the surface for the 25 cm length of the bath before entering a combined drying/drawing module. This took the fiber on a convoluted path through the module where it was dried, and the speed gradually increased until final uptake on the collection spool at 4.00 m min^{-1} (equating to a draw ratio of 320%). This ran for approximately 6 h, producing 1.44 km of fiber. The spools were left overnight to finish drying before being unwound onto cardboard tubes. Before colorimetric analysis, fibers were unwound and rinsed in cold water and air dried. A video file demonstrating the in-flow coloration process is available as part of the ESI.

Color Measurement

Both conventionally dyed and *in-situ* dyed fibers were measured using a Datacolor 500 colour spectrophotometer and analyzed using Datacolor software. From the reflectance (*R*) values at a specified wavelength (λ) of the dyeings, the colour strength (*K*/*S*) of the sample was calculated using the Kubelka-Munk equation (equation 1) below:

$$\frac{K}{S} = \frac{(1-R)^2}{2R} \quad (1)$$

CIELab color data were also obtained by measurement using the Datacolor 500 colour spectrophotometer.

Sustainability Metric Calculations

Energy Intensity was calculated based on the work of Xu *et al.*[1] and Martins *et al.*[2] Values were calculated by equation 2, where Q is the heat added, c is the specific heat capacity of water, m is mass and ΔT is change in temperature.

$$Q = cm\Delta T \quad (2)$$

The following assumptions were made:

- conventional dyeing energy usage assumed 100% efficiency with no extra energy required to maintain temperature during the hold phase, or for the slow ramp speed of 1 °C min^{-1}.
- The system was assumed to start at 20 °C (ambient) prior to ramp.
- No energy consumption from agitation in the conventional dyeing is included in the calculations.
- Because the same fiber (casein) was used in all comparisons, the specific heat capacity of casein was not included in the equation as it would be a constant throughout all calculations.
- *in-situ* energy usage assumed to be 'extra energy' energy required for dyeing, and as there is no dyebath heating ($\Delta T = 0$) and the energy usage for the wet-spinning is unchanged, hence, this is assumed to be negligible.

Material Intensity was calculated based on the work of Xu *et al.*[1] and Martins *et al.*[2] Taking into account all chemicals, dyes and solvents added to the dye bath required to dye 1 kg of fiber. For the *in-situ* system, this included sulfuric acid required as part of the wet-spinning process, dye added, and water. For the conventional dyeing system this included formic acid used to adjust the pH, dye added and water. For literature examples, all additives quoted in the text were included

including any levelling agents. The table below summarizes the material intensity calculations for each dyeing method:

Table S1. Values for calculation of Material Intensity for different coloration methods.

Method	Mass of Water (per kg fiber)	Mass of dye/auxiliary agents (per kg fiber)	Total (Material Intensity)
Coloration in flow	10.0	0.2	10.2
Conventional @ 40 °C	20.0	1.2	21.2
Conventional @ 60 °C	20.0	1.2	21.2
Conventional @ 90 °C	20.0	1.2	21.2
Literature Natural Dyes[3]	40.0	1.2	41.2
Literature Synthetic Dyes[4]	20.0	0.0	20.0

The following assumptions were made:

- *in-situ* water usage: experiment ran the rig for 6 h with no reduction in color (verified by performing colorimetric analysis on fiber samples throughout the run). 1.44 km of fiber

produced = 54 g. 500 mL (0.5 L) dyebath was used and equates to roughly 1:10 fiber-to-liquor ratio equaling a 10 L kg^{-1} water consumption.

- estimates do not include exhaust process in dyeing.

Space Time Yield (STY) is a way of measuring productivity of a flow process in mass per reactor volume per unit time.[5] It is calculated via equation 3 below:

$$STY = \frac{m}{V} = \frac{Product\ Mass\ Flow\ (g\ h^{-1})}{Reactor\ Volume\ (L)} \quad (3)$$

To adapt this metric for use in dyeing, product mass flow is measured as the mass of colored fiber produced per hour and the reactor volume is taken as the dye bath volume.

For the *in-situ* process, the product mass flow was calculated by taking the uptake rate of colored fiber in m h^{-1} and multiplying it by the mass of one m of colored fiber to get a product mass flow in g h^{-1}. This was then divided by the dye bath volume in L (see equation 4).

For conventional batch dyeing, the product mass flow was calculated by dividing the mass of fiber dyed by the total time taken for the dyeing (calculated as the ramp time to take the dye vessel from ambient (20 °C) to the set temperature, plus the dye time). In batch dyeing, the liquor to fiber ratio was set by the experiment, meaning that the dye bath volume is dependent on the mass of fiber

being produced. Therefore, to calculate reactor volume, the mass of fiber dyed in kg was divided by the liquor to fiber ratio in L kg^{-1} to calculate the reactor volume in L. These values were then used to calculate batch space time yield according to equation 5.

$$in\ situ\ STY = \frac{(Colored\ Fiber\ Uptake\ (m\ h^{-1}) \times Mass\ Colored\ Fiber\ (g\ m^{-1}))}{Dye\ Bath\ Volume\ (L)} = \frac{m\ (g\ h^{-1})}{V\ (L)} \quad (4)$$

$$Batch\ STY = \frac{\left({}^{Mass\ of\ Fiber\ Dyed\ (g)} / {}_{(Time\ Taken\ (h))} \right)}{(Mass\ of\ Fiber\ Dyed\ (kg) \times\ Liquor\ to\ Fiber\ Ratio\ (L\ kg^{-1}))} = \frac{m\ (g\ h^{-1})}{V\ (L)} \quad (5)$$

Tables 2 and 3 below give the values used in the calculation of the STY for both the *in-situ* method, and the conventional batch dying methods, from both experimental data and literature values.

Table S2. Values used in STY calculations for coloration in flow.

Method	Fiber Uptake Rate (m h^{-1})	Mass of Fiber (g m^{-1})	Dye Bath Volume (L)	Product Mass Flow (g h^{-1})	STY (g L^{-1} h^{-1})
Coloration in flow	240	0.038	0.05	9.12	182.4

Table S3. Values used in STY calculations for various conventional dyeing methods.

Method	Mass of Fiber Dyed (g)	Time Taken (h)	Liquor: Fiber Ratio (L kg^{-1})	Dye Bath Volume (L)	Product Mass Flow (g h^{-1})	STY (g L^{-1} h^{-1})
Conventional @ 40 °C	5	0.83	20	0.10	6.00	60.0
Conventional @ 60 °C	5	1.17	20	0.10	4.29	42.9
Conventional @ 80 °C	5	1.50	20	0.10	3.33	33.3
Literature Natural Dyes[3]	5 (estimated)	2.33	40	0.2	2.14	10.7
Literature Synthetic Dyes[4]	5 (estimated)	1.28	20	0.1	3.91	39.1

The following assumptions were made:

- STY calculations do not take into account any cooling time required in the batch process.
- STY calculations do not take into account set-up or work-up time required.

- STY calculations are based on information from lab-scale systems for both conventional and *in-situ* dyeing, calculations would need to be performed again on industrial scale to get accurate real-world productivity values.

- STY calculations for the literature examples assumed the same mass of fiber dyed (5 g).

References

(1) Xu, S.; Chen, J.; Wang, B.; Yang, Y. Sustainable and Hydrolysis-Free Dyeing Process for Polylactic Acid Using Nonaqueous Medium. *ACS Sustain Chem Eng* **2015**, *3* (6), 1039–1046.

(2) Martins, A. A.; Mata, T. M.; Costa, C. A. V; Sikdar, S. K. Framework for Sustainability Metrics. *Ind Eng Chem Res* **2007**, *46* (10), 2962–2973.

(3) Benli, H.; Bahtiyari, M. İ. Dyeing of Casein Fibers with Onion Skin-Based Natural Dye Sources after Ozonation. *Ozone Sci Eng* **2018**, *40* (2), 141–147.

(4) Choi, J.; Kim, M. Dyeing Characteristics of Casein Protein Fiber with Acid Dyes and Reactive Dyes. *Textile Coloration and Finishing* **2008**, *20* (5), 14–22.

(5) Polyzoidis, A.; Altenburg, T.; Schwarzer, M.; Löbbecke, S.; Kaskel, S. Continuous Microreactor Synthesis of ZIF-8 with High Space–Time-Yield and Tunable Particle Size. *Chemical Engineering Journal* **2016**, *283*, 971–977.
